# Failure of Acromioclavicular Joint Reconstruction Eight Weeks After Hook Plate Removal: A Case Report

**DOI:** 10.7759/cureus.18640

**Published:** 2021-10-10

**Authors:** Josh A Hansen, John C Dunn, John P Scanaliato, Joshua Caruso, Nata Parnes

**Affiliations:** 1 School of Medicine, Uniformed Services University of the Health Sciences, Bethesda, MD, USA; 2 Orthopaedic Surgery, William Beaumont Army Medical Center, El Paso, USA; 3 Department of Orthopaedic Surgery and Rehabilitation, Claxton-Hepburn Medical Center, Ogdensburg, NY, USA; 4 Department of Orthopaedic Surgery and Rehabilitation, Claxton-Hepburn Medical Center, Carthage, NY, USA; 5 Department of Orthopaedic Surgery and Rehabilitation, Carthage Area Hospital, Carthage, NY, USA

**Keywords:** ac reconstruction, shoulder and elbow, hook plate, orthopaedic surgery, acromioclavicular joint/injuries

## Abstract

A female patient who underwent successful reconstruction of an acute high-grade acromioclavicular (AC) joint separation with hook plate presented with failure of the reduction eight weeks after removal of the hardware. Surgeons and patients should be aware of the risk of late failure of acromioclavicular reconstruction after removal of the hook plate.

## Introduction

Blunt injuries to the acromioclavicular (AC) joint are among the more common injury patterns to the shoulder in athletes and often lead to pain, disability, and associated decreased function and performance [1]. These injuries typically occur from a direct blow to the acromion and result in increasing degrees of AC displacement and associated ligamentous disruption. These injuries are typically classified according to the Rockwood classification system [2]. Types I and II injuries characteristically have minimal AC joint displacement and intact CC ligaments, and these injuries tend to heal uneventfully with non-operative management. Types III-VI are higher energy injuries that result in significant displacement of the AC joint representing complete disruption of the CC ligament complex causing considerable pain, disability, and deformity [1,3]. Types IV-VI are typically treated surgically while the optimal treatment for type III injuries is still hotly debated regarding consensus on optimal treatment [4-5].

Over the years, a wide variety of operative CC ligament reconstructive techniques has been developed to identify an optimal approach to the surgical management of this challenging shoulder injury. The current techniques described are either arthroscopic, open, or combined. Though many of these techniques have demonstrated good results, they each carry with them very specific advantages and disadvantages with regard to risk, patient satisfaction, and functional outcomes [3,6-9]. One of the more commonly described techniques for reconstruction of acute high-grade separation is hook plate stabilization of the AC joint. The technique involves the placement of a superior lateral clavicle plate with a hook portion on the undersurface of the acromion to allow for reduction of the AC joint; however, it requires a second surgery for hardware removal after healing of the coracoclavicular ligaments occurs. Current manufacturer recommendations are for removal of the plate at three months after implantation to allow for adequate healing while minimizing the risk of complications [10]. We present a case report of a 52-year-old female patient who underwent four months of hook plate fixation but demonstrated clinical and radiographic findings of late non-traumatic loss of reduction 8 weeks after hook plate removal.

The patient was informed that data concerning the case would be submitted for publication, and she provided consent.

## Case presentation

**Figure 1:**
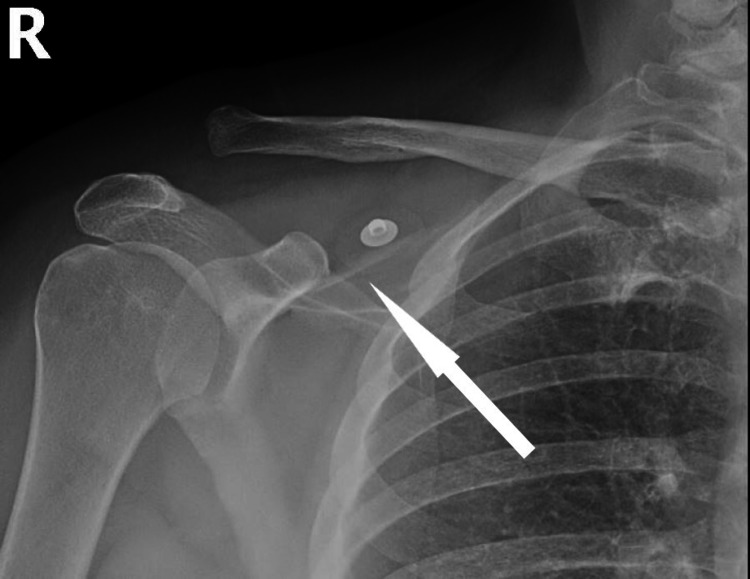
Injury anterior-posterior (AP) X-rays of the patient’s right shoulder at presentation

A 52-year-old female non-smoker with an uncomplicated medical history presented to the orthopedic clinic with right shoulder pain after an all-terrain vehicle (ATV) accident involving a direct blow to the lateral aspect of the shoulder. The injury had occurred the previous day and radiographs from the emergency department revealed a high grade (Rockwood type V) right AC joint separation (Figure 1). In the clinic, her examination confirmed a deformity typical of a type V high-grade AC joint separation with the distal end of the clavicle protruding superiorly and posteriorly. She reported 8 out of 10 on the pain visual analog scale (VAS) and was neurovascularly intact distally about the upper extremity. After a discussion regarding the risks, benefits, and alternatives of treatment, a joint decision was made to treat her injury operatively with a hook plate.

**Figure 2:**
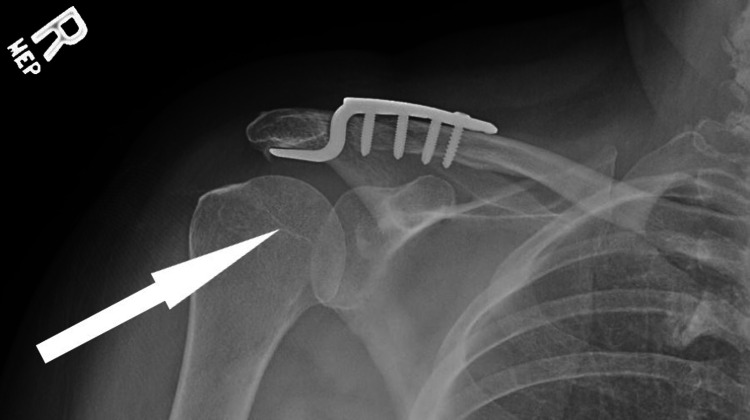
Post-procedural anterior-posterior (AP) X-rays demonstrating right acromioclavicular (AC) reconstruction using a DePuy Synthes hook plate DePuy Synthes, Johnson and Johnson

Fifteen days after her initial injury, the patient was taken to the operating room. She underwent a right shoulder AC joint reduction using a right acromioclavicular four-hole hook plate (DePuy Synthes, Johnson and Johnson, Raynham, MA). A superior approach to the AC joint was used, and the hook of the hook plate and a reduction clamp were used to achieve reduction. The surgery was successfully completed without complications, with the position of the hardware and reduction of the AC joint confirmed by fluoroscopy as well as postoperative radiography (Figure 2). She was transferred to the recovery room and discharged home with instruction for rest and the use of a Gunslinger brace at all times for six weeks.

At six weeks postoperatively, she was removed from the brace and progressed to a course of therapy for the range of motion of the shoulder and self-stretching with continued limitations of heavy use, lifting, pushing, or pulling.

At three months postoperatively, the patient demonstrated maintenance of reduction with a pain-free range of motion of the shoulder, which was symmetric to the contralateral shoulder. Her examination revealed a well-healed incision with no residual tenderness over the AC joint. Active ROM was measured with forward flexion to 160 degrees, external rotation to 70 degrees, and internal rotation to the level of T9. Radiographs at that time demonstrated the appropriate position of the clavicle with near anatomic reduction of the AC joint and some minimal bony reaction on the undersurface of the acromion. The decision was then made to remove the hardware according to the manufacturer’s recommendations to avoid potential complications with the prolonged indwelling of the implant.

**Figure 3:**
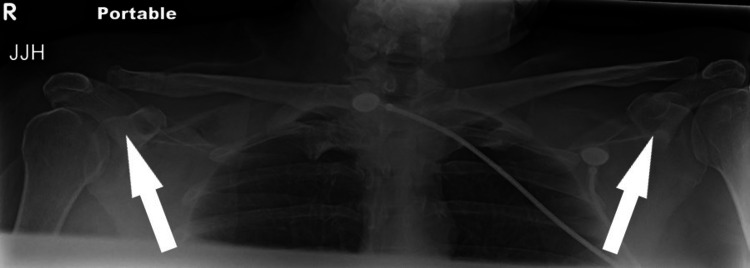
Bilateral anterior-posterior (AP) X-rays after removal of hardware at four months postoperatively

The patient ultimately returned to the operating room four months postoperatively. She underwent removal of the hook plate and associated hardware. The surgery was uneventful, and the AC joint was found to be stable after the removal of the hardware. No loss of reduction was observed intraoperatively, and intraoperative fluoroscopy and postoperative radiographs demonstrated a reduction of the joint (Figure 3). Following surgery, she was transferred to the recovery room and then discharged home the same day with instructions for the use of a shoulder immobilizer for comfort only. After the anesthetic block had worn off, the patient was instructed to begin active-assisted and active ROM exercises and cautioned to avoid overuse or trauma.

Six weeks after the removal of the hardware, the patient returned to the office with a pain score of 0 out of 10. Her exam revealed a well-healed surgical scar, a reduced AC joint with no deformity, and the full range of motion of the shoulder. Her physical therapy was continued with further emphasis on gradual strengthening and stretching.

**Figure 4:**
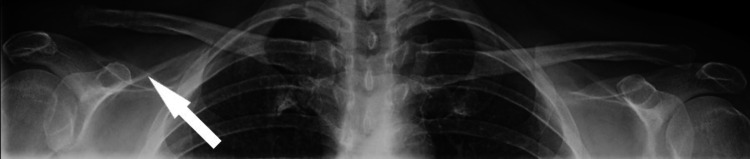
Bilateral anterior-posterior (AP) X-rays upon patient presentation with spontaneous recurrence of right acromioclavicular (AC) deformity and pain at eight weeks after hook plate removal

Eight weeks after hook plate removal, the patient presented with pain and deformity over the AC joint. She denied new trauma and reported a feeling of movement at the clavicle during stretching with the physical therapist. Her examination revealed the clavicle had re-displaced to a posterosuperior position relative to the acromion with point tenderness over the AC joint that was aggravated in cross-chest adduction. Bilateral AC joint radiographs were obtained, which demonstrated a two-fold increase in CC distance on the right side when compared to the left side, confirming the diagnosis of re-separation of the AC joint (Figure 4).

Four weeks later, she underwent revision reconstruction of the AC joint using a semitendinosus tendon allograft. To date, she is six months post-revision and reports a pain score of 0 out of 10, has a subjective shoulder score of 95%, and has resumed her work as a hairdresser.

## Discussion

There are over 150 surgical techniques described for the correction of AC joint separation, but there is no consensus on the ideal fixation method [11]. However, acute high-grade AC joint separation is commonly treated operatively with a hook plate along with strict postoperative rehabilitation guidelines and activity restrictions, with eventual hook plate removal after soft-tissue healing.

The hook plate technique was first introduced in 1976 by Balser [5]. The technique utilizes a contoured plate that mimics the amphiarthrotic nature of the AC articulation while maintaining reduction [12]. The plate is fixed to the superior surface of the distal clavicle and the undersurface of the acromion by an extraarticular hook and functions as an internal splint that maintains an anatomic reduction at the AC joint for the time required to heal the injured CC ligaments [5,13]. In a previous study, 84% of patients treated with a hook plate returned to pre-injury levels and demonstrated excellent functional outcomes [14]. In addition to positive outcomes, the hook plate is widely used because it is known to be a safe, simple, and biomechanically favorable method of fixation [15-17].

Unfortunately, the hook component of the implant can cause bursa and rotator cuff tendon irritation and tears in addition to bony attrition of the acromion, which can lead to acromial fracture [12,18]. As a result of these concerns, it is recommended by the manufacturer that the hook plate be removed after three months, once healing of the CC ligaments has occurred [5,10]. A consequence of such removal is the potential for redislocation. In a cohort of 313 patients, Kienast et al. documented seven redislocations after removal of the hook plate, which is consistent with other reports [14,19-20]. In most cases, failure of the CC ligaments to heal presents as recurrent separation of the AC joint at the time of removal of hardware. This enables the surgeon to perform alternative reconstruction at the time of surgery such as tendon allograft.

Our case is unique due to the delayed failure of the reduction. At the time of hardware removal, there were no radiographic or clinical findings to suggest a failure of ligament healing. However, at eight weeks post hardware removal, the patient experienced a spontaneous recurrence of AC type V separation, suggesting that she had not yet fully healed. It is likely that this patient could have benefitted from a longer fixation period prior to hardware removal, although there were no components of her history or injury to suggest that this was necessary. To date, there are no other reports of spontaneous failure of the AC joint multiple weeks after hardware removal.

## Conclusions

In summary, this patient presented with a late failure of the reduction despite early operative intervention, a successful postoperative course, and relatively delayed removal of the hardware at four months. Based on this case, we suggest that consideration should be given to delayed removal of the hook plate in patients who do not have significant clinical or radiographic evidence of subacromial or rotator cuff irritation or bony attrition, to decrease the possibility of failure and need for further surgical reconstruction.
